# Retraction: Use of Ore-Derived Humic Acids With Diverse Chemistries to Elucidate Structure-Activity Relationships (SAR) of Humic Acids in Plant Phenotypic Expression

**DOI:** 10.3389/fpls.2022.966035

**Published:** 2022-06-16

**Authors:** Frontiers Editorial Office

The journal retracts the 01 December 2021 article cited above.

Following publication, concerns were raised regarding the inclusion of proprietary information in the article. The data in question was generated whilst the corresponding author was working for another commercial company and was included in the article without proper authorization.

This retraction was approved by the Chief Editors of Frontiers in Plant Science and the Chief Executive Editor of Frontiers. All authors agree to this retraction.

